# Macro- and Microphase Separated Protein-Polyelectrolyte Complexes: Design Parameters and Current Progress

**DOI:** 10.3390/polym11040578

**Published:** 2019-03-29

**Authors:** Justin M. Horn, Rachel A. Kapelner, Allie C. Obermeyer

**Affiliations:** Department of Chemical Engineering, Columbia University, New York, NY 10027, USA; jmh2301@columbia.edu (J.M.H.); rak2172@columbia.edu (R.A.K.)

**Keywords:** polyelectrolytes, complex coacervation, protein materials, phase separation, micelles

## Abstract

Protein-containing polyelectrolyte complexes (PECs) are a diverse class of materials, composed of two or more oppositely charged polyelectrolytes that condense and phase separate near overall charge neutrality. Such phase-separation can take on a variety of morphologies from macrophase separated liquid condensates, to solid precipitates, to monodispersed spherical micelles. In this review, we present an overview of recent advances in protein-containing PECs, with an overall goal of defining relevant design parameters for macro- and microphase separated PECs. For both classes of PECs, the influence of protein characteristics, such as surface charge and patchiness, co-polyelectrolyte characteristics, such as charge density and structure, and overall solution characteristics, such as salt concentration and pH, are considered. After overall design features are established, potential applications in food processing, biosensing, drug delivery, and protein purification are discussed and recent characterization techniques for protein-containing PECs are highlighted.

## 1. Introduction

Polyelectrolyte complexation is a process in which oppositely charged polyelectrolytes associate and release bound counterions to decrease the free energy of the solution. Studied by Bungenberg de Jong and coworkers in the 1930′s, the first polyelectrolyte complex (PEC) systems were protein and polysaccharide systems that were found to undergo liquid–liquid (complex coacervation) or solid–liquid (precipitation) phase separation [1]. This phase separation resulted from a two-step process: first, oppositely charged polyelectrolytes associated due to coulombic attraction to form a PEC and second, the entropic gain from bound counterion release drove macroscopic phase separation. The result being macrophase separation to yield two immiscible phases: a polyelectrolyte-rich and a polyelectrolyte-poor phase. The thermodynamic theory of this process was then pioneered by Voorn and Overbeek two decades later [2]. Following these initial experimental and theoretical studies, protein-containing PECs have been studied and applied to a wide range of fields including: food science, drug delivery, protein purification, and enzymatic nanoreactors [3,4,5,6,7,8,9,10,11,12,13,14,15,16].

The interest in and utility of protein–polyelectrolyte macrophase separation spurred subsequent interest in microphase separation. These microphase separated complexes were first demonstrated by Harada and Kataoka in the 1990’s and relied on a block co-polyelectrolyte to induce microphase separation [17]. One block of the polyelectrolyte was an uncharged hydrophilic polymer, while the second block was a charged polymer that was able to complex with an oppositely charged polyelectrolyte to form a net-neutral block. Upon complexation, the block co-polyelectrolyte complex self-assembled into micelles of varying morphologies. Although PECs frequently phase-separate, the interfacial surface tension between the aqueous phase and the coacervate phase is relatively low [18,19,20,21]. The water content in the coacervate phase is also quite high, suggesting a material with diverse solvent interactions, rather than strict hydrophobic interactions that drive assembly and phase separation [22,23]. Protein-containing PEC micelles were demonstrated by Harada and Kataoka in 1998, a few years after their initial demonstration of charge-driven self-assembly [24,25]. This original microphase separated protein PEC incorporated lysozyme as a model protein. Since then, such systems have shown potential as vehicles for intracellular delivery of proteins and polypeptide therapeutics and as nanostorage devices for the long-term protection and storage of proteins [26,27,28,29,30,31,32,33].

**Figure 1:**
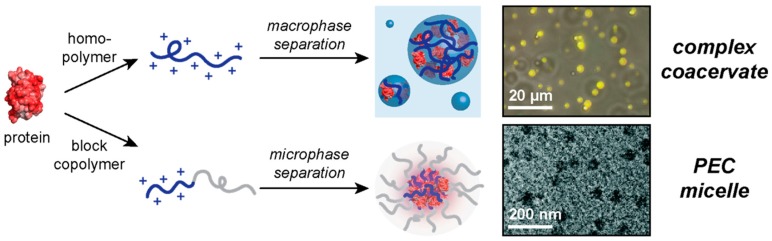
Overview of the content reviewed here. Protein complexation with a polyelectrolyte can lead to macrophase separation when the polyelectrolyte is a homopolymer or microphase separation when the polyelectrolyte is a block copolymer. Fluorescence microscopy image adapted with permission from [34], copyright (2018) American Chemical Society. Electron microscopy image adapted from [35] with permission from The Royal Society of Chemistry, 2016.

In the intervening two decades, an improved understanding of the design principles for protein–polyelectrolyte complexes has been developed. This enhanced understanding has more recently enabled the incorporation of a broader range of non-model proteins in PEC materials. This review covers these recent advances to establish design criteria for incorporation of functional proteins in PECs. We initially focus on macrophase separation to form a complex coacervate phase and then discuss self-assembly to form PEC micelles (Figure 1). Additionally, this review highlights applications of these materials and new techniques to probe the physical properties of PECs. We will outline general design criteria to control the material properties of the PEC phase. For macrophase PECs, this means tuning protein or polymer charge to impact protein partitioning and coacervation or precipitation. For microphase separated PEC micelles, this involves using polymer architecture and external stimuli to control micelle size and morphology, as well as incorporating responsive elements that allow PEC micelles to be optimized for specific applications, such as protein delivery.

## 2. Macrophase Separation of Protein–Polyelectrolyte Complexes

Proteins are weak polyelectrolytes with both positive and negative residues on the solvent-accessible surface. With only five of twenty residues (Asp, Glu, His, Lys, Arg) containing a charge near physiological pH, proteins typically also have relatively low charge density. Despite these significant differences compared to synthetic polyelectrolytes, proteins can participate in macrophase separation by either directly phase separating with an oppositely charged polymer or by partitioning into the coacervate phase formed by two polyelectrolytes.

### 2.1. Protein Design Parameters

The most universal approach to incorporate proteins into PECs relies on protein partitioning into a PEC formed by two other polyelectrolytes. Three-component systems contain a protein of interest in addition to a polycation and a polyanion [3,36,37]. For example, poly-(*N*,*N* dimethylaminoethyl methacrylate) (PDMAEMA) and polyacrylic acid (PAA) were used to incorporate lysozyme into the coacervate phase [36]. This system was optimized to incorporate up to 200 g L^−1^ of lysozyme in the coacervate phase by tuning macromolecule concentrations and the ionic strength of the system [36]. PAA has also been used in combination with poly(allylamine hydrochloride) (PAH) to form coacervates with bovine serum albumin (BSA) [37]. This protein-polymer system was used to identify the importance of the macromolecule mixing order. To maximize incorporation of the protein component, the protein should be mixed first with the oppositely charged polyelectrolyte, as this can result in protein–polymer complexation without macrophase separation. In this case, mixing the negatively charged BSA with PAH prior to addition of PAA increased the concentration of encapsulated BSA [37]. However, it should be noted that if these materials are at equilibrium, protein incorporation should not depend on mixing order. However, due to the relatively low stability of folded proteins, thermal equilibration of PEC materials is frequently not possible. The incorporation of BSA into the coacervate phase has also been studied using the polypeptides poly(lysine) and poly(glutamic acid) where the same mixing order strategy was employed [38,39]. BSA was also efficiently incorporated into these polypeptide based coacervates, demonstrating a relative insensitivity to the precise polymer chemistry. A system containing just a multivalent small molecule (adenosine triphosphate (ATP)) and a polycation (poly(diallyldimethylammonium chloride)) was shown to undergo complex coacervation and successfully sequester enhanced green fluorescent protein (EGFP), as well as other biomolecules [3]. These examples demonstrate that this approach can be used to incorporate native proteins into the complex coacervate phase. However, proteins can also phase separate directly with a polyelectrolyte and this frequently results in a higher protein concentration in the dense phase.

**Figure 2:**
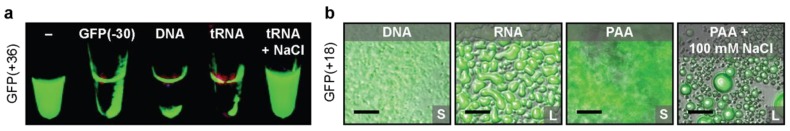
(**a**) Macroscopic phase separation of GFP(+36) with various polyanions. From left to right GFP(+36) with: no polyanion, GFP(−30), dsDNA, tRNA, tRNA + NaCl. Adapted with permission from [42], copyright (2007) American Chemical Society. (**b**) Optical micrographs of macroscopic phase separation of GFP(+18) with various polyanions. From left to right: dsDNA, RNA, PAA, PAA + 100 mM NaCl. Adapted with permission from [41], copyright (2017) American Chemical Society.

Protein design parameters that facilitate the direct complexation and phase separation with a polyelectrolyte are currently being studied. Due to the Coulombic nature of the interactions, the primary focus has been on factors that influence electrostatic attraction, including macromolecule charge and solution ionic strength. One protein design parameter that has been studied is the importance of overall protein charge on phase separation. While some naturally occurring proteins, such as BSA, have been shown to phase separate directly with a polyelectrolyte (such as hyaluronic acid), many do not [40]. The effects of overall protein charge on the ability of a protein to phase separate with a polyelectrolyte has been studied by chemically modifying several globular proteins with succinic anhydride to systematically decrease the net protein charge [35]. By increasing the ratio of negative to positive residues on the protein, phase separation could be induced with model polycations. Further increases to this ratio allowed for efficient partitioning of the protein into the coacervate phase [35]. The effect of protein supercharging was also studied by making point mutations to the model protein superfolder GFP (sfGFP) to increase the ratio of positive to negative residues [41,42]. Increasing the net number of basic residues on sfGFP resulted in variants that phase separated with polyanions, including RNA and DNA (Figure 2a) [42]. It was also determined that increasing the net charge on supercationic GFP increased the critical salt concentration for phase separation with a range of synthetic and biological polyanions (from 150 mM to 3 M) [41]. Additionally, it was observed that low salt concentration frequently resulted in precipitation of the cationic GFPs and polyanions, but the amorphous precipitate transitioned to a liquid coacervate upon the addition of salt (Figure 2b).

**Figure 3:**
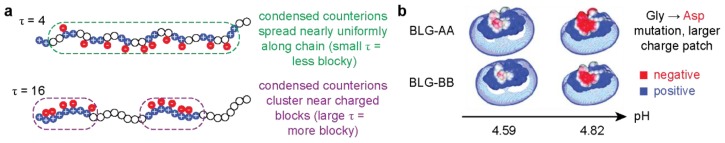
Polyelectrolyte and protein charge patchiness are key factors in macrophase separated complexes. (**a**) Visual representation of the distribution of counterions on charge patches of different sizes. Figure adapted from [43] under open access license. (**b**) Visual representation of negative charge patches on two different BLG mutants at increasing pH. Adapted with permission from [44], copyright (2011) American Chemical Society.

Proteins are amphoteric, weak, sequence-specific polyelectrolytes that often adopt a globular folded structure. Therefore, in addition to the overall charge, the distribution of charges on the protein can also influence complexation and phase separation with polyelectrolytes. One systematic way of studying the effect of charge distribution on complex coacervation is to use polypeptides as they can be synthetized by solid phase peptide synthesis and provide a simpler model scaffold as compared to a globular protein [43]. In a study by Chang et al., the complex coacervation of poly(glutamate) and sequence-defined poly(glycine-*co*-lysine) with varied 1D patterns of charged monomers, was investigated experimentally and computationally [43]. It was determined that patterns of charges affect the binodal phase boundary and the critical salt concentration. Polypeptides with longer stretches of charged residues (large *τ*) remained phase separated at higher salt concentrations due to the increased entropy gain from the release of a greater number of counterions condensed on the longer charged block (Figure 3a) [43].

Phase separation does not necessarily require the protein to be supercharged; proteins with sufficient charge density or a localized charged patch have been shown to undergo complex coacervation as well [45]. The ionizable nature of charged amino acids (Asp, Glu, His, Lys, Arg, Tyr) allows a charge patch to be introduced simply by altering the pH of the system [46]. Charge patches on proteins can be identified using the protein crystal structure. Software such as Pymol (with the APBS plugin) and DelPhi can be used to calculate and visualize the electrostatic surface potential of a protein [42,46,47]. For example, the charge patch that develops on β-lactoglobulin (BLG) at its isoelectric point is sufficient to induce phase separation with gelatin B [48]. The charge patch that forms on BSA near its pI was exploited to selectively phase separate BSA from a mixture of BSA and BLG with the ampholytic polypeptide gelatin B [16]. Even more impressively, Dubin and co-workers have shown that the difference a single point mutation (Gly → Ala) confers to the size of the charge patch on the BLG dimer was sufficient to enable selective complex coacervation of the mutated isoform (Figure 3b) [44].

If a charge patch is not identified on the protein of interest at the desired pH, the protein can also be engineered to have a specific charge patch. Recently, several sfGFP variants were engineered to be net neutral, but with different charge distributions, with one variant designed to be a Janus particle with positive and negative charge localized to opposite sides of the protein [47]. The cationic charge patch was thought to facilitate interaction of this GFP variant with anionic cartilage [47]. As an alternate approach, a charge patch has also been introduced to sfGFP by engineering mutants with an anionic polypeptide tag on the C-terminus [49]. These polypeptide tags served to supercharge sfGFP while also localizing the negative charge to a single site on the protein. This system demonstrated that the addition of a charge dense tag increases the critical salt concentration of GFP/quaternized poly(4-vinyl-*N*-methylpyridium iodide) (qP4VP) coacervates over those formed using a GFP mutant of equal charge, but with the charge evenly distributed across the globular domain [49]. In combination, these studies of synthetic polypeptides as well as native and engineered proteins highlight the importance of the spatial arrangement of charges with a larger “patch” of like-charged residues promoting complexation and phase separation.

The addition of a polyionic tag to a globular protein not only introduces a charge patch, but also adds an unstructured domain. Proteins that undergo simple coacervation naturally, such as mussel foot proteins (MFPs) and elastin like polypeptides (ELPs), tend to have unstructured, intrinsically disordered regions (IDRs) [21,50,51,52,53,54,55,56,57]. Native proteins that phase separate can de-mix by simple coacervation, but frequently the conditions under which phase separation occurs can be expanded via complex coacervation with an oppositely charged biopolymer. For example, MFP-1 undergoes complex coacervation with hyaluronic acid [21,50]. This ability has increased interest in developing recombinant mussel foot proteins for various complex coacervate-based applications, such as bio-based adhesives [50]. Some proteins, such as Whi3 and LAF-1 not only have regions of disorder but also have RNA binding domains that facilitate complexation and phase separation with RNA [58,59]. Proteins with IDRs, particularly those that interact with RNA are often components of membraneless organelles; it is hypothesized that one of the driving forces for their formation is complex coacervation [60,61,62]. Complex coacervation is also hypothesized to be one of the driving forces behind the condensation of DNA on the protein histone H1, which has a disordered C-terminus [63]. A recent study by Turner et al. looked at the effect of phosphorylation at three serine residues in the C-terminal domain (CTD). It was concluded that increasing the negative charge on the CTD by post-translational modification decreased partitioning of DNA into the coacervate phase, consistent with complex coacervate behavior [63].

Finally, heteroprotein systems that undergo complex coacervation are also of great interest to the field [48,64,65,66]. These multi-protein coacervates are particularly relevant to the formation and biological function of membraneless organelles [67,68]. In this case, the protein design parameters, particularly intrinsic disorder and protein charge anisotropy, are important factors for heteroprotein phase separation [64].

These recent efforts to determine the factors that govern protein complex coacervation have opened new avenues for these materials. Motivated by the potential applications and enabled by improved understanding of protein macrophase separation with polyelectrolytes, many proteins have been incorporated in macrophase separated coacervates (Table 1). While examples of the incorporation of therapeutic or catalytic proteins remain limited, the scope of protein coacervates is rapidly increasing. To date, proteins of varying size (10–150 kDa) and charge (−28 to +189) have successfully been complexed with polyelectrolytes to form complex coacervates.

### 2.2. Polymer Design Parameters

In addition to the protein component, the polyelectrolyte partner also plays a significant role in determining the phase boundary and the likelihood of liquid–liquid (L–L) or solid–liquid (S–L) phase separation. There are three main categories of polymers used in protein complex coacervate systems: synthetic polymers, bio-based polymers, or a complimentary protein. Synthetic polymers have the advantage of increased chemical diversity, which can be used to vary the backbone or impart orthogonal functionality, but can suffer from a lack of biocompatibility as well as lack of control over polymer length and monomer placement. Common synthetic polycations studied in protein complex coacervate systems include qP4VP, PDMAEMA, PAH, and poly(ethylenimine) (PEI) [35,36,37,41,83]. One concern when designing polycations for complex coacervate materials, particularly for biological applications, is their cytotoxicity. Polyesters have been used as the inspiration for the design of some biocompatible polycations. A betaine functionalized polyester was designed to act as a biocompatible polycation for complex coacervate applications [84]. Poly(arginine) based polyesters, such as poly(ethylene argininylaspartate diglyceride) (PEAD), have also been studied for this same reason [85,86]. Common synthetic polyanions complexed with proteins include PAA and poly(styrene sulfonate) (PSS) and they do not appear to be plagued by issues of cellular toxicity [37,41]. However, bioinformatics analysis of the proteomes from several organisms indicates that native proteins are biased toward net negative charge indicating that polyanions may have less utility for the complexation of unmodified proteins.

To address the limitations of synthetic polyelectrolytes for protein coacervates, significant effort has been dedicated to studying biological or bio-based polymers. In fact, bio-based polymers were used in the initial reports on complex coacervation [1]. They are good candidates for a range of complex coacervate applications as they are generally biocompatible. Bio-based polymers that have been studied include heparin [87] and chitosan [88], as well as polypeptides, such as poly(lysine) [43,75,89], poly(arginine) [71] and poly(glutamic acid) [89]. Bio-based polyanions are of particular interest for complex coacervate applications with proteins because they are thought to be key components of complex coacervates that form in vivo. These include single stranded (ssDNA) and double stranded DNA (dsDNA) [41,63,85,90,91] as well as RNA [41,60,84,92,93]. RNA and DNA also allow the direct investigation of the impact of linear charge density (via base pair hybridization) on phase separation [90]. Anionic polysaccharides, such as hyaluronic acid, have also been extensively studied due to their biocompatibility and prevalence in food and personal care applications [21,40]. For example, pectin has been used to study how polyelectrolyte charge density impacts phase separation by varying the degree of methyl esterification of the carboxylic acids on pectin [83,88,94,95].

Upon phase separation, the protein-containing dense phase can be a liquid, a viscoelastic gel, or an amorphous precipitate [77,89,96]. Designing a system that creates the desired condensed phase upon phase separation is, therefore, of critical importance. While the design factors that result in S–L or L–L phase separation are still relatively unknown, there are several hypotheses currently being investigated, many of which have explored the role of the polymer component.

One hypothesis is that the nature of phase separation can be directed by the macromolecule mixing ratio. This is of particular interest in heteroprotein systems [48,76]. Varying the mixing ratio for a BLG, BSA, gelatin B system resulted in either L–L phase separation or gelation. By zeta potential measurements, it was determined that the L–L systems were observed closer to a charge neutral state than the gel systems. It was observed that in the L–L system, the gelatin molecules were in the coil state but in the gel system, they were in the helix state [76,77]. Similar changes in the nature of phase separation have also been observed in polyelectrolyte–protein systems [41,86,97]. Decreasing the relative amount of GFP to polyanion resulted in a transition from S–L to L–L phase separation [41].

Polypeptide systems have been used to study whether S–L phase separation occurs due to the formation of a secondary structure. Polypeptides have been used to study the effects of chirality on the nature of phase separation. It has been observed in a system using poly(lysine) and poly(glutamic acid) that polypeptides with eight or more sequential L-chiral residues will undergo S–L phase separation with β-sheets formed in the precipitate phase [98]. Systems containing fewer than eight sequential L-chiral residues or one racemic peptide underwent L–L phase separation, and no β-sheets were formed [98,99]. The formation of secondary structure within the coacervate in this simplified system mimics that observed with gelatin B and globular proteins.

DNA is a common polyanion used in protein coacervate studies on the nature of phase separation. It has been shown in a system with poly(l-lysine) that the hybridization state of DNA impacts the nature of phase separation; ssDNA undergoes L–L phase separation while dsDNA undergoes S–L phase separation [90]. Vieregg et al. hypothesized that this behavior is observed due to dsDNA having a higher charge density than ssDNA [90]. This behavior has also been observed in PECs containing a globular protein. It has been shown that RNA undergoes liquid–liquid phase separation, while dsDNA undergoes solid–liquid phase separation with engineered cationic GFPs [41].

In addition to charge (ratios, density), polymer architecture can also affect protein–polyelectrolyte de-mixing. Advances in controlled polymer synthesis have enabled the preparation of charge matched linear and comb polyelectrolytes. The effect of polymer architecture was studied using both comb and linear poly(lysine) and poly(glutamic acid) [100]. It was determined that the comb poly(lysine) underwent liquid–liquid phase separation with both stereoregular and racemic poly(glutamic acid), while the linear poly(lysine) underwent solid–liquid phase separation with the stereoregular poly(glutamic acid) and liquid–liquid phase separation with the racemic poly(glutamic acid) [100].

Finally, the strength of the interactions between the protein and polyelectrolyte is also thought to govern the nature of phase separation [40,96]. A study by Comert et al. looked at phase separation as a function of ionic strength and pH using BLG and a monoclonal antibody with hyaluronic acid and tragacanthin [40]. Potentiometric titrations and turbidimetry were used to conclude that increasing the magnitude of the charge patch on the protein would increase the strength of the interaction between the protein and the polyelectrolyte and favor S–L phase separation [40,96]. A detailed examination of these factors is detailed in a recent review [96]. These findings indicate that the geometry and strength of interactions, as well as the potential for secondary interactions, influence the nature of the second phase. Taken to an extreme, the polymer architecture can also be tuned to create microphase separated particles with either a liquid-like or solid-like core.

## 3. Microphase Separation of Protein–Polyelectrolyte Complexes

### 3.1. Polymer Design Parameters

By simply substituting a block copolymer polyion for the polyelectrolytes discussed in the previous section, protein PECs self-assemble to form a homogeneous solution of microphase separated particles. Generally, two components are required to create a microphase separated polyelectrolyte complex: a block copolymer with a polyelectrolyte block to drive self-assembly and an oppositely charged polyelectrolyte to complex with the polyelectrolyte block copolymer. The polyelectrolyte block can be paired with either a hydrophilic or hydrophobic neutral block [101]. The hydrophobicity of this neutral block determines whether the polyelectrolyte complex is located in the core or the corona of the micelle.

If paired with a hydrophobic neutral block, the block copolymer will form a micelle even in the absence of the oppositely charged polyelectrolyte [102]. Such micelle-forming block copolymers have been used to complex a variety of polyelectrolytes, including nucleic acids [103,104], polymeric polycations [102], and proteins [105]. In one example, poly(dimethylaminoethyl methacrylate)-*block*-poly(styrene) (PDMAEMA-*b*-PS) micelle-forming copolymers were complexed with PSS homopolymers [102]. Well-defined, hydrophobic core micelles formed when either the polycation or polyanion was in significant excess, as monitored by dynamic light scattering (DLS) and cryogenic electron microscopy (cryo-EM). However, mixing ratios near charge neutrality, resulted in the formation of large, multi-micellar aggregates. This was proposed to be due to the corona becoming net-neutral, coacervate-like, and hydrophobic, resulting in aggregation and macrophase separation of the micelles. While this strategy has been successfully applied to incorporate charge-dense biological polyanions such as nucleic acids, proteins are less commonly incorporated into these systems. Given the differences in behavior, constitutive material, and applications, this review will focus on PEC micelles that have the coacervate phase in the core of the micelle, surrounded by a hydrophilic corona.

To create a PEC core micelle, a di-block copolymer with a charged block and a neutral, hydrophilic block is combined with an oppositely charged polyelectrolyte (or neutral-charged block copolymer). At polymer molar ratios that approach overall charge neutrality, the polyelectrolytes will complex and condense into a complex coacervate. Driven by favorable electrostatic interactions and counterion release, soluble polyelectrolyte complexes first form. Near overall charge neutrality, concentration of these soluble complexes is sufficient to cause aggregation into a microphase-separated micellar structure. This microphase-separated micelle is stabilized and shielded from solvent interactions by the neutral block, resulting in microphase separation of a consistent morphology and size.

**Figure 4:**
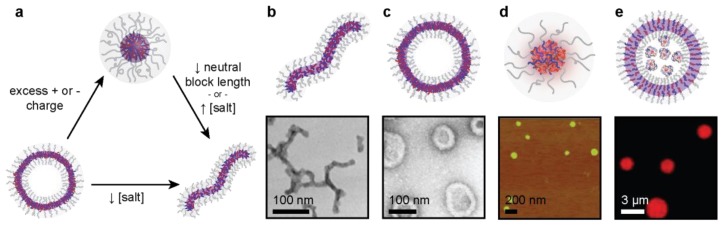
(**a**) Stimuli-induced transitions in PEC microphase morphology. Microphase morphology has experimentally transitioned between spherical micelles (top), cylindrical micelles (bottom right), and vesicle (bottom left) when subject to the listed stimulus. (**b**) TEM images of cylindrical PEC micelles from PEG-*b*-p(Asp-AP) and homo-p(Asp). Adapted with permission from [119], copyright 2014 American Chemical Society. (**c**) TEM images of PEC vesicles from homo-p(Asp-C5) and PEG-*b*-p(Asp). Adapted with permission from [32], copyright 2014 American Chemical Society. (**d**) AFM image of spherical PEC micelles from CytC and PEG-*b*-pAsp(DET). Adapted with permission from [27], copyright 2009 John Wiley and Sons. (**e**) Confocal laser scanning microscopy image of PEC vesicles from PEG-*b*-p(Asp-AP), PEG-*b*-p(Asp), filled with myoglobin. Adapted with permission from [123], copyright 2007 John Wiley and Sons.

A variety of morphologies have been reported, including spherical micelles [17,24,25,106,107,108,109], worm-like micelles [106,110,111,112], vesicles [32,113,114,115,116,117], and lamellae [106,118,119,120,121]. Micelle morphology is influenced by a variety of factors, depending on the particular polymers, including salt concentration [106,112], mixing ratio [107,110], temperature [119,122], and relative size of each component of the system [119] (Figure 4).

In the case of salt concentration, morphological changes result from two factors: swelling of the core of the micelle, and change in solubility of the hydrophilic block. Micellar swelling stems from solvation of the core due to increased screening of electrostatic interactions [112]. For example, poly[sodium 2-(acrylamido)-2-methylpropanesulfonate]-*block*-poly[2-(methacryloyloxy)ethyl phosphorylcholine] (PAMPS-*b*-PMPC) anionic-net neutral block copolymers and poly{[3-(methacryloylamino)propyl]trimethylammonium chloride}-*block*-PMPC (PMAPTAC-*b*-PMPC) cationic-net neutral block copolymers were combined at a constant total macromolecule concentration and charge ratio. For both polymers, the PMPC block is zwitterionic, but net neutral. Single-phase solutions at a positive charge ratio of 0.6 were formed with an initial sodium chloride concentration of 2 M. Sodium chloride concentration was decreased until microphase separation was observed by optical microscopy. For all tested solutions, phase separation occurred between 1 M and 0.4 M NaCl. Micelle morphology was also observed via small angle x-ray scattering (SAXS). Between 0.5 M and 0.05 M NaCl, bilayer vesicles were observed, and below 0.02 M NaCl, cylindrical micelles were observed. In summary, at high salt, a single-phase system was observed. Decreasing salt concentration led to phase-separated PEC vesicles. A further decrease in salt concentration caused a phase transition to cylindrical micelles [124]. Similar transitions between vesicle and micelle morphologies have been observed with other polymers. Specifically, micelles transitioned from spherical to cylindrical due to increasing salt concentration [112]. Microphase separated PECs from PAA and poly(*N*-methyl-2-vinylpyridium-*block*-poly(ethylene oxide) (PM2VP-*b*-PEO) were characterized by SAXS, dynamic light scattering (DLS), and static light scattering at varying concentrations of NaCl. As salt concentration increased, the micelle core swelled with solvent, while the PEO corona shrank in size due to decreasing solvent (water) solubility. These two factors combined to cause a spherical to worm-like transition in micelle morphology [112]. These two representative examples illustrate the complex relationship between salt concentration and morphology of the microphase separated polymers.

Morphological transitions can also be induced by changing the mixing ratio of the cationic and anionic components [107,110]. In the PAMPS-*b*-PMPC/PMAPTAC-*b*-PMPC polymer system mentioned previously, the system was found to create vesicles at a salt concentration of 0.1 M NaCl and a positive charge fraction of 0.55. However, at charge fractions of 0.4 and 0.8 there was excess positive or negative charge in the core of the micelle. This resulted in a morphological transition from vesicles to spherical micelles. The transition occurred to minimize electrostatic repulsion in the charged core [107,110]. These factors have also been observed to tune the nature of the condensed phase in macrophase separation and are key design parameters for the preparation of any protein–polyelectrolyte complex. However, some variables that tune the morphology and size of these self-assembled particles are specific to the block copolymer architecture required for microphase separation.

Changing the relative size of the hydrophilic neutral block also results in morphological transitions. PEG-*block*-poly([5-aminopentyl]-α,β-aspartamide) (PEG-*b*-p(Asp-AP)) was added to a mixture of PEG-*block*-poly(α,β-aspartic acid) (PEG-*b*-p(Asp)) and homo-poly(α,β-aspartic acid) (p(Asp)) to form microphase separated PECs. The PEG weight fraction was varied from 6.5% to 12.1%, while keeping the length of the charged blocks constant. As the quantity of the hydrophilic PEG block was decreased, micelle morphology transitioned from spherical to worm-like to lamellar. This is consistent with previous results and was due to a change in the relative sizes of the hydrophilic and complexed blocks [112]. Here, an analogy can be drawn to the Israelachvili packing parameter for surfactant micelles [112,125]. Increasing the length of the hydrophilic chain is analogous to increasing the size of the hydrophilic head group in a surfactant micelle. The result is an increase in the packing parameter, which shifts micelle morphology from spherical to cylindrical to lamellar. Additionally, PEG-*b*-p(Asp-AP)/p(Asp) was also tested for thermal sensitivity. Increasing temperature tunes the PEG-solvent interactions, resulting in the PEG adopting a less swollen state with increasing temperature. This decrease in PEG volume fraction resulted in cylindrical, connected cylindrical, or ordered lamellar morphologies, depending on the overall weight fraction of PEG [119].

Like micelle morphology, micelle size is also affected by properties of the constitutive molecules and environmental conditions such as salt concentration, pH, and temperature. In most systems, micelle size is primarily determined by the length of the charged block of the block copolymer. Specifically, van der Kooji et al. determined that, for a PAA/PM2VP-*b*-PEO system, micelle size was unaffected by PAA size below a certain critical threshold [112]. This threshold is related to the point at which a single PAA homopolymer is sufficient for overall charge neutrality of the micelle. Above this point, micelle size increases with PAA homopolymer size. Below this threshold, size increases with PM2VP block length due to an increase in micellar aggregation number [108,112]. The hydrophilic block also has a small influence on micelle size, specifically by increasing the size of the corona.

In addition to polymer length, solution conditions can also affect micelle size. In the PAA/PM2VP-*b*-PEO system, salt was found to have a complicated effect on overall micelle size. Increasing salt concentration can also impact the micelle size in two ways: swelling due to increasing core solvation and decreasing the aggregation number. Therefore, depending on which of these factors is most important for a given system, salt will either increase or decrease the overall micelle size. If smaller homopolymers are used, the core swelling effect is dominant and micelle size increases. When larger homopolymers are used, the decrease in aggregation number is dominant and micelle size decreases [112]. In microphase PECs composed of three or more polyelectrolyte components, the relationship becomes even more complex. In general, polyelectrolytes with a lower charge density are more affected by the electrostatic screening of salt ions. This can change microphase composition (and therefore size) by causing less charge-dense polyelectrolytes to preferentially leave the PEC microphase. For example, spherical PEC micelles composed of poly(acrylic acid)-*block*-poly(acrylamide) (PAA-*b*-PAAm) and lysozyme were compared to three component micelles composed of PAA-*b*-PAAm, PDMAEMA, and lysozyme. Increasing salt concentration caused core swelling and an increase in size in the two-component micelles. However, the three component micelles saw a decrease in micelle size. This was, attributed to expulsion of the less charge-dense lysozyme from the core of the micelle in favor of the smaller homopolymer/copolymer micelles [126].

A final factor in PEC micelle design is their colloidal stability with respect to key stimuli, such as salt concentration [32,105,108,112,126,127], pH [26,27,117,128], temperature [32,117,119], polyelectrolyte concentration [119], and reducing agents [78]. As PEC microphase separation is driven by complexation of oppositely charged polyelectrolytes, PEC micelles will disassemble at charge fractions far from charge neutrality. If either charged polyelectrolyte is in significant excess, instead of a microphase separated micelle, the polyelectrolytes will form soluble complex particles [109]. In addition, like all micelles, PEC micelles have an overall critical micellization concentration below which the micelles will disassemble [102]. Because PEC micelles are formed through electrostatic interactions, PEC micelles also have a critical salt concentration above which they disassemble. This is due to screening of electrostatic interactions in the core of the micelle and is analogous to salt-induced disassembly of bulk phase coacervates [126,127]. As an example, the PAA-*b*-PAAm, PDMAEMA, lysozyme system studied by Lindhoud et al. was tested for salt stability by monitoring micelle size by DLS as a function of salt concentration. NaCl concentration was gradually increased until micelles could no longer be detected [73]. This dissolution due to charge screening is a common feature of coacervate systems and is critical to many potential applications of these materials.

### 3.2. Protein Design Parameters

Though primarily tested on synthetic polymer systems to date, many of the behaviors and design rules for PEC microphases also apply to protein-containing PEC micelles. Such protein-containing PEC micelles have many potential applications as nanocarriers for protein therapeutics, enzymatic nanoreactors, and protein protectors and stabilizers. Proteins are most commonly incorporated into PEC micelles as a complimentary homopolymer, alongside a neutral-charged block copolymer. In one such example, Lee et al. used synthetically modified, anionic equine heart cytochrome (CytC) with PEG-*block*-poly[*N*-{*N*′-(2-aminoethyl)-2-aminoethyl}aspartamide (PEG-*b*-p(Asp-DET)) to create PEC micelles for intracellular CytC delivery [27]. In this case, the negatively charged CytC took the place of an anionic homopolymer in driving complexation and micelle formation. Additionally, depending on the chemical modification method, the PEC micelles could be made responsive to endosomal pH, facilitating the cytosolic delivery of CytC. A complementary incorporation strategy involves a three component system, whereby an existing synthetic polymer PEC micelle system is used as a nanocarrier for a charged protein. If the charged protein has the same charge as the synthetic homopolymer, it can preferentially partition into the micelle microphase [129].

The protein component does not have to participate as a “homopolymer”. The covalent attachment of a hydrophilic, neutral polymer to the protein results in a bioconjugate that when combined with a polyelectrolyte can create PEC micelles. In such systems, the polymer-decorated protein serves as the neutral-charged block copolymer, complexing an oppositely charged homopolymer to drive micelle formation. For example, Jiang and co-workers attached poly(oligo (ethylene glycol) methyl ether methacrylate) to BSA (POEGMA-*g*-BSA). The BSA bioconjugate was then used in two separate systems to form PEC micelles with lysozyme or Sprouty 1. Because of the soluble POEGMA block, the normally unstable protein–protein complexes were able to form stable PEC micelles that ultimately facilitated intracellular protein delivery [130]. This pioneering example has several potential implications for the development of protein PEC micelles. This approach could provide a straightforward way to co-deliver several therapeutic proteins. Additionally, these protein-based PEC micelles could be used to selectively filter proteins of interest. In polymer di-block PEC micelles, it has been shown that polyelectrolytes selectively phase-separate with polyelectrolytes of similar charged block length [131]. The extension of this chain length recognition phenomenon to ampholytic, globular proteins remains an open question.

The protein components of PEC micelles follow similar design criteria to the polymer components. They must have sufficient charge density to drive complexation, be appropriately sized relative to the other components, and be combined with another polyelectrolyte at a ratio at or near overall charge neutrality. But because proteins usually take the place of the homo-polyelectrolyte, the protein component has little influence on the overall size or morphology of the micelle.

The most important factor in selecting a protein to incorporate into a PEC micelle is the charge of the protein at the pH of interest. The protein must be sufficiently charged to allow for complexation and self-assembly of the micelle. A wide variety of proteins have been incorporated into PEC micelles including: lysozyme [26,126,127,132,133,134], BSA [78,130], GFP [135,136,137,138], and glucose oxidase (Table 2) [139]. These include proteins with a naturally high surface charge such as lysozyme and BSA. In cases where the natural charge of the protein is not sufficient to promote complexation and self-assembly, synthetic modifications can be performed to convert cationic amino acids to anionic residues. These modifications can have the advantage of adding a responsive element that allows for controlled changes in the micelles. For example, immunoglobulin G (IgG) antibodies, reversibly modified with citraconic anhydride, were combined with a PEG-*b*-p(Asp-DET) block copolymer. At physiological pH (7.4), PEC micelles were formed. At endosomal pH (5.5), the citraconic anhydride-modified moieties cleave to reveal the original cationic side chains. The result is that a protein that would not normally microphase separate with a neutral-charged block copolymer can do so to create a PEC micelle that is responsive to acidic pH [30].

This charge-conversion strategy can also be applied to the polymer component to create a pH responsive micelle. Changes in pH result in a charge-conversion from negative-to-positive and thus change the overall charge ratio, causing disassembly of the micelles at acidic pH values [26,27]. In one example from Wu et al., a poly(ethylene glycol)-*block*-poly[(*N*′-citraconyl-2-aminoethyl)aspartamide] PEG-*b*-p(Asp-EDA-Cit)/lysozyme PEC micelle was prepared. The p(Asp) block contains an unstable anionic citraconic amide. At acidic pH, the citraconic amide degrades into a cationic primary amine. As a result of this degradation, these micelles dissociated within two hours at pH 5.5. This pH-induced degradation allowed for uptake and controlled release of active, unmodified lysozyme at physiologically relevant conditions [26].

Like their macro-phase separated counterparts, PEC micelle formation is driven by electrostatic interactions. Salt screens these electrostatic interactions and, at a critical salt concentration, can therefore induce dissolution of the PEC micelle. Salt effects are particularly relevant for protein containing PEC micelles, as the lower charge density of proteins makes them particularly susceptible to charge screening [129]. This can be advantageous, as the protein component can be preferentially removed from the micelle without full dissolution of the micelle. For example, poly(2-methyl vinylpyridinium)_41_-*block*-poly(ethylene oxide)_205_ was combined with PAA_139_ and lipase and compared to PEC micelles composed of just the polymers without lipase. The micelles were titrated with increasing concentrations of sodium chloride and the size and dispersity were monitored by DLS and small angle neutron scattering (SANS). Based on changes in micelle size as a function of salt concentration, it was determined that, by a salt concentration of 0.12 M NaCl, all the lipase had been removed from the micelle. Full disintegration of the micelles did not occur until 0.5 M NaCl for either system [129]. As the ability to design either macro- or microphase separated complexes with any protein of interest improves, new potential applications of these materials are made possible and additional functional characterization techniques are required.

## 4. Recent Development of Applications and Characterization Techniques

### 4.1. Applications of Protein–Polyelectrolyte Complexes

**Figure 5:**
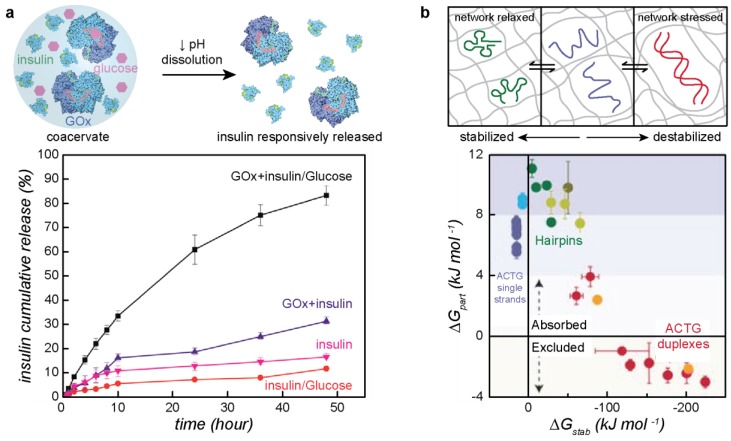
Applications of macrophase separated coacervates. (**a**) Controlled release of insulin from the coacervate phase for insulin delivery. Insulin and glucose oxidase are coencapsulated into the coacervate phase. Glucose will partition into the coacervate phase and react with the glucose oxidase to produce gluconic acid. This decrease in pH results in dissolution of the coacervate phase and the release of insulin. Reprinted with permission from [34], copyright (2018) American Chemical Society. (**b**) An example of using complex coacervates as a molecular sieve. Protein coacervates containing Ddx4 can selectively partition different types of DNA and RNA by destabilizing the nucleic acid structure. ssDNA and RNA as well as DNA and RNA with a hairpin conformation will partition due to their relative instability whereas DNA and RNA duplexes will be excluded. Reprinted with permission from [145], copyright (2016) Springer Nature.

Protein complex coacervates have numerous applications due to their ease of preparation. To date, one of the most common uses of protein coacervates is their use in food products to stabilize small molecules [4,5,6,7,8,143]. They are also being explored as a method to co-localize proteins to act as bioreactors [9,10]. In addition, appropriate design of coacervate system could allow for protein complex coacervation to be used as a protein purification technique [12,16,144] or as a means of protein immobilization for surface coating [52]. Finally, macrophase separated systems are being explored as a method for the delivery of biomolecules [3,13,14,15]. A recent study that combines these two applications, conducted by Lim et al., looked at encapsulating glucose oxidase and insulin in the coacervate phase for controlled insulin release (Figure 5a) [34]. The concentration of glucose controls the rate of insulin release. When glucose partitions into the coacervate phase, the glucose oxidase converts it to gluconic acid. The resulting decrease in pH results in the dissolution of the coacervate phase and the subsequent release of insulin [34].

Since complex coacervation is also hypothesized to be one of the driving forces of membraneless organelle formation, protein complex coacervates are also being explored for applications that mimic their evolved cellular functions. In a recent study by Nott et al., the partitioning of different DNAs and RNAs in droplets of phase separated Ddx4 was quantified (Figure 5b) [145]. It was concluded that the protein network formed upon phase separation affected the partitioning of different nucleic acids and could thus be used as a molecular filter to exclude double stranded sequences. Due to the differences in partitioning of the DNA and RNA and the proteins tested, it is hypothesized that these molecular filters can be used as in vivo reactors [145].

**Figure 6:**
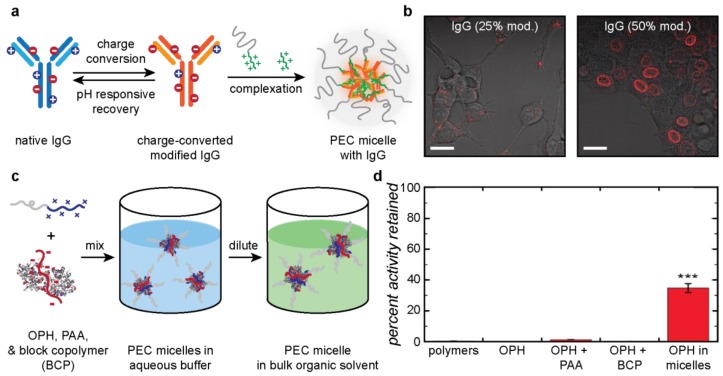
Applications of microphase separated protein–polyelectrolyte complexes. (**a**) Schematic for charge conversion and formation of IgG-containing PEC micelles. (**b**) Observation of in vitro IgG PEC micelle delivery to C26 murine colon carcinoma cells after 24 h incubation. (left) IgG-25/m; (right) IgG-50/m. Adapted with permission from [30], copyright 2016 American Chemical Society. (**c**) Schematic of encapsulation of organophosphate hydrolase in PEC micelles for protection against small molecule denaturants. (**d**) Percent activity retained by OPH against methyl paraoxon after 24 h incubation in DMMP. Adapted with permission from [140], copyright 2016 American Chemical Society.

Similarly to macrophase separated droplets, PEC micelles can protect proteins from degrading agents to maintain protein activity [142] and secondary structure, and deliver proteins intracellularly via endocytosis [27,29,30,31,123]. The most widely studied application of microphase separated protein PECs involves the use of PEC micelles as nanocarriers for intracellular delivery of protein therapeutics. These micelles have reasonable colloidal stability in vivo and, through judicious choice of macromolecular components, can be made to respond to changes in pH, temperature, or the presence of reducing agent. Such changes allow for controlled disassembly of the micelle upon delivery into the cell. As a result, PEC micelles represent a versatile platform for controlled delivery of many different proteins with careful control of the point of release. For temperature responsiveness, a polymer with a physiologically relevant LCST is used to confer sensitivity. For example, poly[2-(dimethylamino)ethyl methacrylate]-*block*-poly(glutamic acid) (PDMAEMA-*b*-p(Glu)) was found to disassemble at basic pH when cooled below 40 °C due to a PDMAEMA LCST around this temperature [146]. Such temperature responsiveness has not yet been explored for protein-containing PEC micelles, but represents an interesting potential area for development. Conversely, pH-responsiveness through the presence of weak or charge-conversional polyelectrolytes is a common feature in intracellular, PEC micelle delivery systems. One such example used charge-conversional antibodies (IgG) that bind to a family of nucleoporins and a PEG-*b*-p(Asp-DET) block copolymer to form pH-responsive PEC micelles [30]. The IgG was functionalized with citraconic anhydride to convert IgG into a polyanion. The charge-converted IgG micelles were assayed for the effectiveness in delivering IgG to the nuclear envelope of C26 cells. Micelles were taken into the cell through endocytosis and, at endosomal pH, the citraconic anhydride moieties were cleaved, exposing the original cationic side chains of the protein. This resulted in disassembly of the micelle and localized delivery of IgG to the nuclear envelope, as shown by fluorescence microscopy (Figure 6a). Importantly, the degree of IgG modification had a significant effect on the rate of the charge reversal and the degree of endocytosis. These factors were balanced with 50% modification of side chain amines, resulting in efficient cellular uptake and endosomal release [30].

Micelles formed via polyelectrolyte complexation have been used in several non-delivery related applications including catalytic systems [32,147], stabilization and nanostorage [140,142], and to create protein microarrays [148]. In terms of nanostorage, proteins can be incorporated into PEC micelles without significant decrease in activity, while protecting the protein from possible denaturants [140]. In one example of stabilization and nanostorage, PAA and poly(oligo(ethylene glycol) methacrylate)-*block*-poly(4-vinyl N-methylpyridyl iodide) (POEGMA-*b*-qP4VP) were used to create PEC micelles that encapsulated organophosphate hydrolase (OPH) [140]. The OPH, when encapsulated, was shown to be stabilized in organic solvents and resistant to degradation from dimethyl methylphosphonate (DMMP), a simulant of the organophosphate class of nerve agents. Further, after disassembly of the micelles, the OPH was found to retain 35% of its original activity. OPH alone, in the presence of a block copolymer, or mixed with PAA homopolymer retained less than 5% of original activity after incubation with DMMP. Similarly, when incubated in a different organic solvent (ethanol), the OPH containing PEC micelles retained 88% of the original OPH activity compared to a maximum of 48% retained activity for OPH alone (Figure 6b) [140].

### 4.2. Characterization Techniques for Macro- and Microphase Separated Protein–Polyelectrolyte Complexes

**Figure 7:**
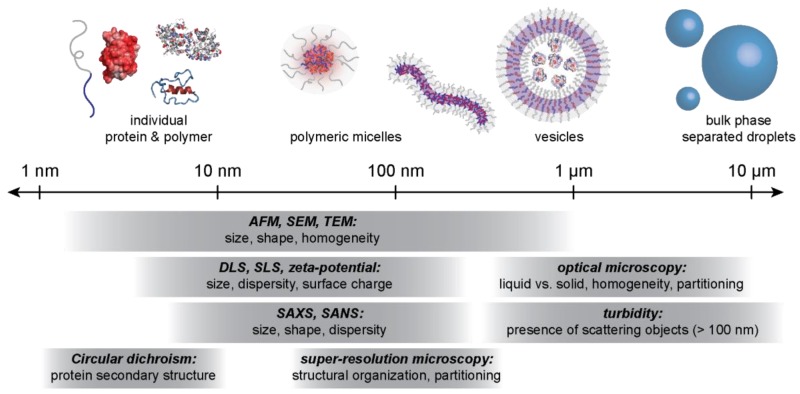
Common characterization and imaging techniques for protein–polyelectrolyte complexes. Protein–polyelectrolyte complexes span several length scales. The combination of several analytical techniques can be used to characterize the constituent molecules, micellar assemblies, and macrophase separated droplets.

As the ability to design either macro- or microphase separated polyelectrolyte complexes with any protein of interest continues to improve, new potential applications of these materials are made possible and additional functional characterization techniques are required. There are many different techniques to analyze and characterize complex coacervates (Figure 7). Turbidity measurements are often used as an indicator of macrophase separation [35,37,38,41,45]. DLS can be used as an indicator of macrophase separation as well as give additional insight into the size of the droplets [46,149,150]. Light scattering techniques such as DLS and static light scattering (SLS) are also common techniques to characterize PEC micelles. Small angle scattering techniques (SANS and SAXS), have been used to characterize micelle and droplet morphology [74,77]. Zeta potential measurements have been used to identify the optimal macromolecule ratio for phase separation by determining when the complexes approach charge neutrality [48,80,81,149,150]. The nature of phase separation can be characterized using microscopy. Optical microscopy is commonly used to identify the nature of macrophase separation while transmission electron microscopy has the resolution (<0.2 nm) to resolve the nature of phase separation in the core of a PEC micelle [32,35,41,49,74,77,119,151]. Analyzing the protein component in the protein–polyelectrolyte complexes is also of great importance. Circular dichroism has been used to show that proteins encapsulated in the coacervate phase maintain their secondary structure [28,37,38,51,99,105,150,152]. These standard characterization techniques are thoroughly covered in several recent reviews, thus we will only highlight biophysical techniques that have more recently been applied to protein-based PECs [153,154,155,156].

Fluorescence recovery after photobleaching (FRAP), coupled with optical microscopy, has been used to characterize the dynamics of the coacervate phase in macrophase separated systems; the apparent diffusion coefficient of the macromolecules in the coacervate phase can be derived from FRAP data [82]. Rheology has also been used to characterize the dynamics and relaxation of the condensed phase, as well as quantify the linear viscoelasticity of the material [48,77,149,157,158]. Both of these techniques, however, are bulk level measurements that assume Fickian diffusion. A technique recently developed to analyze the transport properties within a coacervate is super-resolution microscopy based on stimulated emission depletion coupled with fluorescence lifetime correlation spectroscopy (STED-FLCS) [79]. STED allows for visualization on the length scale of the coacervate network (nm) and can be used to measure small molecule diffusion of a spot size below the diffraction limit of light [79]. STED-FLCS has been used in a study by Shakya et al. to probe the dynamics of DNA/poly-l-Lysine droplets using several fluorescent dyes. It was concluded that some small molecule (fluorophore) diffusion in these droplets is non-Fickian [79]. This new technique will help the field advance by understanding the diffusion of various small molecules in the coacervate phase and enabling their use in controlled release applications.

The dynamics of PEC micelles have also recently been monitored by fluorescence-based techniques. Förster resonance energy transfer (FRET) has been used to study the dynamics of PEC micelle self-assembly. For example, Nolles et al. examined PEC micelles, containing *Aequorea Victoria*-derived fluorescent proteins (such as green fluorescent protein, SYFP2, and mTurquoise 2) [135,136,137,138]. In one study, micelles containing FRET-paired proteins, mTurquoise2 and SYFP2, and a cationic-neutral block copolymer poly(2-methyl-vinyl-pyridinium)_128_-*block*-poly(ethylene-oxide)_477_ were examined. FRET was used to determine the kinetics of micelle formation. Results indicated that micelles fully formed after about 600 s and kinetics of formation were first order with respect to the free fluorescent protein concentration. Protein exchange between pre-formed micelles was also monitored as a function of time. Within 200 s, the micelles had reached equilibrium concentrations. Both processes were first order with a relaxation time of 100 s at low ionic strength [135]. That both protein exchange and formation were first order implies that PEC micelle formation and equilibration both involve the exchange of smaller, near-neutral charge protein polymer complexes.

## 5. Conclusions and Outlook

**Scheme 1:**
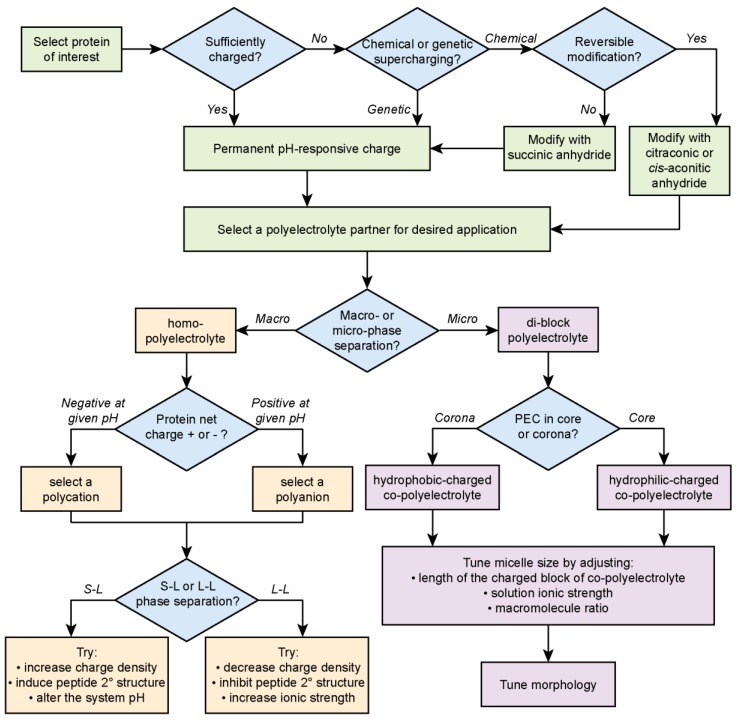
Flow chart for the design of protein–polyelectrolyte complexes.

Protein-containing polyelectrolyte complexes are an exciting class of materials with a range of applications in the food, personal care, and biomedical industries. New avenues of research have demonstrated the potential of complex coacervates to responsively deliver high value biologics, including insulin and monoclonal antibodies [30,34,141]. Additionally, the relevance of protein–polyelectrolyte complex coacervates to the organization of cellular contents via membraneless organelle formation underscores the need to understand the formation and function of this important class of materials. The potential utility of these phase separated materials has spurred recent efforts to develop design criteria for the formation of macro- and microscopic phase separated complexes. Beginning with the protein of interest, several key design decisions lead to the final desired material morphology (Scheme 1).

Most importantly, the protein net charge dictates both the decision to modify the protein synthetically or genetically and the selection of an appropriate polyelectrolyte or pair of polyelectrolytes. More recently, the significance of protein charge “patchiness” on the complexation and binodal phase boundary has been uncovered. This provides another opportunity to tune the behavior and stability of these materials [47,49]. Advances in polymer synthesis have enabled the preparation of “patchy” charged polymers containing neutral, zwitterionic, or ampholytic co-monomers. In combination with protein patchiness, this provides a rich design space to develop advanced polyelectrolyte complex materials [43,159]. In particular, the use of patchy polyampholytes and polyelectrolytes has great potential for protein-containing coacervates as the polymer component can potentially be tuned to match the protein surface chemistry [160]. Advances in the knowledge of the factors that govern protein–polyelectrolyte complexation and phase separation have enabled a range of proteins to be incorporated in these materials (Table 1 and Table 2). However, to date PECs have still primarily been limited to model proteins, such as fluorescent proteins. One limiting factor arises from the focus on the influence of electrostatics on complexation. While electrostatic interactions are the predominant factor governing the formation of these materials, significant differences in behavior are observed between PECs formed with seemingly similar polyelectrolytes. The influence of the polymer backbone, ion-containing functional group, monomer sequence, and polymer architecture on PECs will enable the determination of how additional molecular interactions (hydrophobic, dipolar, cation–π, π–π) contribute to PEC behavior. Understanding the subtle impact of these factors may help to expand the fraction of the millions of known proteins that can be incorporated into PEC materials [161].

Expanding the scope of protein–PEC materials also requires an improved ability to predict how protein incorporation in the polyelectrolyte material affects protein stability and activity. To achieve this lofty goal, we will need to determine how protein sequence and polymer chemistry impacts both protein incorporation in the PEC as well as the protein stability and activity in the condensed phase. This will require methods to measure macroscopic dynamics, (macro)molecular diffusion, and protein structure within the polyelectrolyte complex. Recent reports have demonstrated the potential of fluorescence-based methods adopted from the biophysical community to provide new insights for protein–PEC materials [79,135,162]. Continued multidisciplinary interaction between biophysicists and polymer scientists has great potential to facilitate advances in the functional and dynamic characterization of protein-based PEC materials [73]. Ultimately, predictive design criteria for the formation and function of protein–polyelectrolyte complexes will be required to advance the biotechnological applications of these materials.

## Figures and Tables

**Table 1:** Summary of proteins incorporated in bulk complex coacervate phases.

Protein	Molecular Weight (kDa)	pI	Appx. Charge ^1^	Polyelectrolyte Partners ^2^
α-Chymotrypsinogen ^3^	25.6	8.7	4	PDMAEMA [35]; qP4VP [35]
Actin	42.1	5.4	−13	pLK and pRE [69]
β-lactoglobulin	19.9	5.0	−7	hyaluronic acid [40];tragacanthin [40]; lactoferrin [46]; gelatin B [48]; pectin [70]
BSA	69.3	6.2	−12	PAA and PAH [37]; pLK and pRE [38]; hyaluronic acid [45]; pDK [71]; gelatin B [72]; gelatin B and β-lactoglobulin [16,73]; RPC-bPEI [74]
C-terminal histone 1	11.2	11.2	45	dsDNA [63]
GFP	~27.8	6.6	−7	PDDA and ATP [3]; qP4VP [49]; PEI [49]; PLL [49]
supercharged GFP	~28	-	+36 to −30	RNA [41,42]; DNA [41,42]; PAA [41]; PSS [41]; supercharged GFP [42]; qP4VP [49]; PEI [49]; PLL [49]
glucose oxidase	63.3	5.1	−28	insulin and DgHBP-2 [34]
Histone	30–70	N/A	N/A	ssDNA [75]
LAF-1	76.3	7.1	−2	RNA [58]
lysozyme	14.3	7.3	8	PDMAEMA [35] ^3^; qP4VP [35] ^3^; PAA and PDMAEMA [36]; RPA-bPEI [74]; ovalbumin [76]; pectin [77]; HAMA and CSMA [78]
mfp-1	108	10.0	189	hyaluronic acid [52]; MADQUAT [56]
monoclonal antibody	145	8.3	N/A	hyaluronic acid [40]
myoglobin ^3^	17.1	7.8	0	PDMAEMA [35]; qP4VP [35]
Ovalbumin	42.7	5.3	−12	Pectin [79]; lysozyme [76]
RNase ^3^	16.5	8.6	6	PDMAEMA [35]; qP4VP [35]
tau	78.9	6.7	−7	RNA [80]
Whi 3	71.3	8.4	4	RNA [59]
zein	26.5	6.2	2	dsDNA [81]; Pectin [82]

^1^ The approximate protein charge of the unmodified protein was determined using isolated side chain pKa values and the Henderson-Hasselbalch equation. ^2^ Abbreviations used here: ATP: adenosine triphosphate; BSA: bovine serum albumin; CSMA: chondroitin sulfate methacrylate; Dg-HBP-2; histidine rich humboldt squid beak protein; DNA: deoxyribonucleic acid; dsDNA: double stranded DNA; GFP: green fluorescent protein; HAMA: hyaluronic acid methacrylate; MADQUAT: poly(2-dimethylamino)ethyl methacrylate) methyl chloride quaternary salt; PAA: poly(acrylic acid); PAH: poly(allylamine hydrochloride); PDDA: poly(diallyldimethylammonium chloride); PDMAEMA: poly(*N*,*N*-dimethyl aminoethyl methacrylate); pDK: poly-d-lysine; pLK: poly-l-lysine; pRE: poly-d,l-glutamic acid; PSS: polystyrene sulfonate; qP4VP: poly(4-vinyl *N*-methyl pyridinium iodide); RNA: ribonucleic acid; RPA-bPEI: branched polyethyleneimine reducible polyanion; RPC-bPEI: branched polyethyleneimine reducible polycation. ^3^ These proteins were used in a modified form with an unspecified charge.

**Table 2:** Summary of proteins incorporated in polyelectrolyte complex micelles.

Protein	Molecular Weight (kDa)	pI	Appx. Charge ^1^	Polyelectrolyte Partners ^2^
α-Chymotrypsinogen	25.6	8.7	4	PEG-*b*-p(Asp) [33]; POEGMA-*b*-q4VP and PAA [140]
equine heart cytochrome ^3^	11.8	9.6	9	PEG-*b*-p(Asp-DET) [27]; PEG-*b*-p(Asp-EDA-Suc) [27]
glucose oxidase	63.3	5.1	−28	PEG-*g*-PAA [139]
human serum albumin	66.5	6.2	−11	PEG-*b*-PAE-*c*-API [28]
immunoglobulin g (IgG) ^3^	170	7.4	Natively 0.89	PEG-*b*-p(Asp-DET) [141]
lipase (Humicola lanuginosa)	31.8	5.6	−7	P2MVP-*b*-PEO and PAA [129,142]
lysozyme	14.3	7.3	8	PEG-*b*-p(Asp-EDA-Cit) [26]; POEGMA-*b*-qP4VP [35] ^3^; PAA-*b*-PAAm and PDMAEMA [126,127]; P2MVP-*b*-PEO and PAA [129]; POEGMA-*g*-BSA [130]; mPEG-*b*-p(L-Asp) [133]; PEG-*b*-p(Asp) [25,132,134]
myoglobin ^3^	17.1	7.8	0	POEGMA-*b*-q4VP [35]
organophosphate hydrolase	39.0	8.9	4	POEGMA-*b*-qP4VP and PAA [140]
RNase ^3^	16.5	8.6	6	POEGMA-*b*-qP4VP [35]
Sprouty 1	35	8.7	8	POEGMA-*g*-BSA [130]
**Fluorescent Proteins**				
EGFP	26.9	6.0	−7	P2MVP-*b*-PEO [137,138]
mEGFP	27.0	6.1	−7	P2MVP-*b*-PEO [136]
mCherry	26.7	6.5	−6	P2MVP-*b*-PEO [136]
mKO2	24.5	5.9	−9	P2MVP-*b*-PEO [136]
mTurquoise2	26.9	5.8	−8	P2MVP-*b*-PEO, SYFP2, SBFP2, and mTurquoise2 [135]; P2MVP-*b*-PEO [136]
SBFP2	26.8	6.2	−7	P2MVP-*b*-PEO, SYFP2, SBFP2, and mTurquoise2 [135]; P2MVP-*b*-PEO [136]
SYFP2	26.9	6.1	−7	P2MVP-*b*-PEO, SYFP2, SBFP2, and mTurquoise2 [135]; P2MVP-*b*-PEO [136]
TagRFP	26.1	7.5	0	P2MVP-*b*-PEO [136]

^1^ The approximate protein charge of the unmodified protein was determined using isolated side chain pK_a_ values and the Henderson-Hasselbalch equation. ^2^ Abbreviations used here: mPEG-*b*-p(L-Asp): poly(ethylene glycol)-*block*-poly(L-aspartic acid sodium salt); P2MVP-*b*-PEO: poly(2-methyl-vinyl-pyridinium)-*block*-poly(ethylene oxide); PAA: poly(allyl amine); PAA-*b*-PAAm: poly(acrylic acid)-*block*-poly(acrylamide); PDMAEMA: poly(*N*,*N*-dimethyl aminoethyl methacrylate) oligoethylene glycol methacrylate); PEG-*g*-PAA: PEG-*graft*-poly(acrylic acid); PEG-*b*-PAE-*c*-API: PEG-*block*-poly(β-amino ester)-*co*-1-(3-aminopropyl) imidazole; PEG-*b*-p(Asp): PEG-*block*-poly(aspartic acid), with various ⍵-end groups; PEG-*b*-p(Asp-DET): PEG-*block*-poly[*N*-{*N*′-(2-aminoethyl)-2-aminoethyl}aspartamide]; PEG-*b*-p(Asp-EDA-Cit): PEG-*block*-poly[(*N*′-citraconyl-2-aminoethyl)aspartamide]; PEG-*b*-p(Asp-EDA-Suc): PEG–*block*-poly[(*N*′-succinyl-2-aminoethyl(aspartamide)]; POEGMA-*b*-qP4VP: poly(oligoethylene glycol methacrylate)-*block*-poly(4-vinyl *N*-methyl pyridinium iodide). ^3^ These proteins were used in a modified form with an unspecified charge.
